# The efficient physiological strategy of a novel tomato genotype to adapt to chronic combined water and heat stress

**DOI:** 10.1111/plb.13339

**Published:** 2021-10-04

**Authors:** S. Francesca, L. Vitale, C. Arena, G. Raimondi, F. Olivieri, V. Cirillo, A. Paradiso, M. C. de Pinto, A. Maggio, A. Barone, M. M. Rigano

**Affiliations:** ^1^ Department of Agricultural Sciences University of Naples “Federico II” Portici Italy; ^2^ Department of Biology, Agriculture and Food Sciences National Research Council Institute for Agricultural and Forestry Systems in the Mediterranean Portici Italy; ^3^ Department of Biology University of Naples “Federico II” Naples Italy; ^4^ BAT Center ‐ Interuniversity Center for Studies on Bioinspired Agro‐Environmental Technology Portici Italy; ^5^ Department of Biology University of Bari “Aldo Moro” Bari Italy

**Keywords:** Combined stress tolerance, heat stress, limited water availability, novel genotypes, Reduced Representation Sequencing, *Solanum lycopersicum*

## Abstract

- Climate change is increasing the frequency of high temperature shocks and water shortages, pointing to the need to develop novel tolerant varieties and to understand the mechanisms employed to withstand combined abiotic stresses.
- Two tomato genotypes, a heat‐tolerant *Solanum lycopersicum* accession (LA3120) and a novel genotype (E42), previously selected as a stable yielding genotype under high temperatures, were exposed to single and combined water and heat stress. Plant functional traits, pollen viability and physiological (leaf gas exchange and chlorophyll *a* fluorescence emission measurements) and biochemical (antioxidant content and antioxidant enzyme activity) measurements were carried out. A Reduced Representation Sequencing approach allowed exploration of the genetic variability of both genotypes to identify candidate genes that could regulate stress responses.
- Both abiotic stresses had a severe impact on plant growth parameters and on the reproductive phase of development. Growth parameters and leaf gas exchange measurements revealed that the two genotypes used different physiological strategies to overcome individual and combined stresses, with E42 having a more efficient capacity to utilize the limiting water resources. Activation of antioxidant defence mechanisms seemed to be critical for both genotypes to counteract combined abiotic stresses. Candidate genes were identified that could explain the different physiological responses to stress observed in E42 compared with LA3120.
- Results here obtained have shown how new tomato genetic resources can be a valuable source of traits for adaptation to combined abiotic stresses and should be used in breeding programmes to improve stress tolerance in commercial varieties.

## Graphical abstract

One tomato line was identified that has a high genetic variability and carries key traits for adaptation to combined water and heat stress.

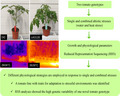

## INTRODUCTION

Plants are continuously subjected to many abiotic and biotic stresses, from seed germination throughout the whole life cycle (Deryng *et al*. 2014), which are now intensified by climate change. In particular, water and heat stress are two of the most critical abiotic stresses limiting crop growth and productivity worldwide, especially in arid and semi‐arid areas (Vitale *et al*. 2009; Arena *et al*. 2020). In the past few years, the combination of high temperature and water scarcity has caused global losses in crop production id ˜US$30 billion (Gupta *et al*. 2020). Improving crop production under water limitation (Farooq *et al*. 2009) and elevated temperatures (Wahid *et al*. 2007) is therefore a primary goal in agriculture (Rigano *et al*. 2016). There are many factors, including high temperature, high light intensity and dry winds, which can increase evaporation of water from the soil and lead to drought. These factors can also increase water loss from plants and, consequently, enhance plant exposure to water stress leading to crop yield reductions (Trenberth *et al*. 2014; Nankishore & Farrell 2016). Furthermore, it has been shown that at temperatures above 35 °C, both the formation and viability of pollen are highly compromised, causing an additional reduction in final yield (Olivieri *et al*. 2020). The simultaneous occurrence of high temperature and soil water depletion may result in a range of morphological, anatomical, physiological and biochemical adjustments in plants in order to counteract these constraints (Chaves & Flexas 2009). Plant responses to these abiotic stresses and the extent of damage vary depending on species, growth stage and the severity of the stress applied (Fahad *et al*. 2017).

One of the physiological processes most sensitive to water and heat stress in plants is photosynthesis. Indeed, under prolonged drought, reduced stomatal conductance limits CO_2_ uptake, and heat stress might also affect biochemical reactions of the photosynthetic machinery (Zhou *et al*. 2017a), limiting plant carbon gain (Hussain *et al*. 2019). It is demonstrated that drought causes photoinhibition of photosystem II (PSII) (Arena *et al*. 2008; Vitale *et al*. 2008), leading to a reduction in both photosynthetic electron chain functionality and Rubisco activity (Zhou *et al*. 2018a, 2018b), but can also trigger mechanisms of damage repair (Murata *et al*. 2007; Arena *et al*. 2008). A further important consequence of abiotic stresses is the alteration to the oxidative metabolism, which causes the accumulation of reactive oxygen species (ROS), such as hydrogen peroxide (H_2_O_2_). Hydrogen peroxide, due to its high permeability across membranes and long half‐life, can also work as a molecular signal able to activate downstream pathways with protective effects (de Pinto *et al*. 2015). However, ROS production beyond a threshold value can lead to oxidative stress, through protein oxidation and lipid peroxidation of cellular membranes, breakdown of photosynthetic pigments and decreased enzyme activity (Sánchez‐Rodríguez *et al*. 2016; Zhou *et al*. 2019; Demirel *et al*. 2020). The accumulation of antioxidant compounds contributes to prevent oxidative damage and lipid peroxidation and thereby to protect cell membrane (Zhou *et al*. 2019; Francesca *et al*. 2020). Moreover, in order to counteract the production of ROS, antioxidant defence mechanisms are normally activated, which involve the action of enzymatic antioxidants including ascorbate peroxidase (APX), peroxidase (POD) and catalase (CAT) and non‐enzymatic antioxidants such as ascorbic acid (AsA) and glutathione (GSH) (Zhou *et al*. 2020b).

Tomato (*Solanum lycopersicum* L.) is one of the most cultivated vegetable crops worldwide. It has an optimal growth diurnal temperature range of 25–30 °C and is known to be sensitive to both water shortage and heat, although its sensitivity varies among genotypes (Rigano *et al*. 2016; Zhou *et al*. 2018b; 2020b; Arena *et al*. 2020; Francesca *et al*. 2020). Climate change is exacerbating and/or increasing the frequency of high temperature shocks and water shortages in the Mediterranean Basin, pointing to the need to develop varieties with enhanced tolerance to naturally co‐occurring stresses (Zhou *et al*. 2020a,b). Indeed, despite the comprehensive literature on plant responses to single stresses, the response to multiple stresses is rarely addressed. In this regard, novel tolerant genotypes should be identified to improve the traditional varieties and also to investigate physiological mechanisms controlling tolerance to combined abiotic stresses. Previously, we identified in our laboratory one tomato genotype (E42) from an arid and warm region of southern Italy that had high and stable yields when grown under high temperatures in open fields (Olivieri *et al*. 2020). However, the analyses of tolerance traits in a controlled environment are often necessary in order to reduce the complexity of interactions between genetic and environmental effects on plant phenotypes and to clearly define the onset of abiotic stress (Zhou *et al*. 2018b). The aim of the present study was to confirm the observed tolerance to elevated temperatures of E42 by performing a rigorous phenotyping under controlled conditions in comparison with one known heat‐tolerant genotype (LA3120). Moreover, we further investigated the responses of these two genotypes to limited water availability and combined water and heat stresses. This study allowed us to analyse the different strategies activated in the two genotypes in response to single and combined stresses. Finally, the genetic variability of these genotypes was investigated by exploiting available Reduced Representation Sequencing (RRS) data. RRS is a powerful tool to identify thousands of DNA polymorphisms, thus greatly reducing the sequencing costs. Among RRS, Restriction Site‐Associated DNA sequencing (RAD‐seq), for example, is highly cost‐effective and can score 200,000 single nucleotide polymorphisms (SNPs) with the same coverage depth as Whole Genome Sequencing of the same quantity of individuals (Scheben *et al*. 2017). Altogether, the phenotypic and genotypic data points recorded in the present work were used for the identification of candidate genes potentially associated with tolerance to abiotic stresses in the two analysed genotypes. Results here reported can lead to further understanding of the response to combined abiotic stresses, a quite common condition in agricultural systems, and could be used for the selection and breeding of tolerant tomato genotypes able to maintain stable crop production in the most critical production areas.

## MATERIAL AND METHODS

### Plant material and experimental design

One tomato genotype selected at the University of Naples, Department of Agricultural Sciences, named E42, and one heat‐tolerant tomato accession, named LA3120 (Tomato Genetics Resource Centre, TGRC, University of California, CA, USA) were used in this work. Both genotypes have a determinate growth habit. The genotype LA3120 is characterized by medium‐sized fruits (70–100 g), while the E42 genotype is characterized by small ‘cherry’ fruits. Seeds were sown in seed trays and, after 20 days, the seedlings were transferred to plastic pots (21‐cm diameter) with commercial substrate in two controlled growth chambers located at the Department of Agricultural Science, University of Naples (Italy). The climate settings of the chambers were 29/24 °C and 16 h/8 h photoperiod in the control chamber, while in the hot chamber the temperatures were 35/30 °C with a 16 h/8 h photoperiod. Plants were grown in a completely randomized block design with three replicates per genotype and five plants per treatment in each replicate. The experiment included four treatments:

1. Control: 29/24 °C, 100% irrigation
2. Water deficit: 29/24 °C, 50% irrigation
3. Heat stress: 35/30 °C, 100% irrigation
4. Combined stresses: 35/30 °C, 50% irrigation

The treatments were applied on 30‐day‐old seedlings and lasted for 3 weeks. The climate control was supported by the Arduino Mega 2560 micro‐control system. The temperature of the two rooms was measured by the DHT11 sensor every 5 min and the data were saved on the SD (Secure Digital) slot. The automatic irrigation system was based on nine soil moisture sensors in pots in each room. Micro‐flow irrigation was applied using self‐compensating 4 l · h^−1^ drippers. The percentage humidity (% v/v) of the substrate in the 100% and 50% irrigation treatments was calculated by averaging measurements taken from at least five pots. The irrigation intervention was carried out when the average percentage water content in the substrate fell below 20%. For the 100% and 50% irrigation treatment, 34 and 17 ml of water per pot were delivered, respectively. After a pause of 30 min, the Arduino system read the average value of the soil moisture sensors for each chamber and, if the average value of the readings for each chamber was less than the set point, it activated the irrigation pump again for 15 min. The system was set up to provide a maximum of three irrigations per day per single chamber. The software was written in Arduino IDE (native environment for Arduino programming).

### Plant biomass and leaf functional trait determination

Plants at mature fruiting stage were harvested 3 weeks after stress treatments and were separated into shoots and roots by cutting at the cotyledonary node. Shoot fresh weight (FW) was determined and all leaves from each plant were counted. Root FW was determined, and the roots cleaned and weighed. The root/shoot ratio was calculated as FW of root/FW of shoot. FWs and number of red ripe fruits were also determined. The leaf functional traits, namely leaf area (LA) and specific leaf area (SLA), were determined on five well‐exposed and fully expanded leaves per genotype per treatment, following the methods of Cornelissen *et al*. (2003). For the measurements, the fourth leaf from the apex in each plant was chosen. The LA was measured using the program Image J 1.45 (Image Analysis Software) and expressed in cm^2^. The SLA was calculated as the ratio of LA to leaf dry mass (cm^2^ · g^−1^). Leaf dry mass (DW) was obtained by drying the leaves in an oven at 70 °C for 48 h.

### Pollen viability

Pollen viability was analysed using three flowers per plant sampled from three different plants per replicate. Pollen grains were spread on microscope slides and one drop DAB solution (Sigma‐Aldrich, Darmstadt, Germany) was added to each pollen sample; slides were gently warmed with a gas lighter and mounted with a cover slip (Dafni 1992). Scoring was made under an Leitz Laborlux12 microscope. This easy to perform test was chosen altough pollen viability based on benzidine may not always be correlated to pollen germination (Janssen & Hermsen 1976).

### Leaf gas exchange and chlorophyll *a* fluorescence

Leaf gas exchange and chlorophyll fluorescence were simultaneously performed by using the Li6400 portable photosynthesis system (LiCor, Lincoln, NE, USA) integrated with Li6400‐40 Leaf Chamber Fluorometer, which acts as both a leaf cuvette and light source/pulse‐amplitude modulated fluorometer. Measurements were carried out in the morning (09:00–11:30 h) on fully expanded mature leaves with the following environmental parameters: photosynthetic photon flux density of 1000 μmol photons m^−2^ · s^−1^, 360 μmol CO_2_ mol^−1^, relative humidity 50–55%,and two fixed temperature regimens: 25 °C (considered as ambient control) and at 35 °C (considered as heat treatment). Net photosynthesis (*A_N_*), stomatal conductance (*g_s_*) and transpiration (*E*) were calculated according to von Caemmerer & Farquhar (1981) with the software of the Li6400. Steady‐state fluorescence (*F_s_*) and maximum fluorescence ($F_{m}^{\prime }$) upon illumination were measured after a 0.8‐s saturating flash of 7000 μmol photons m^−2^ · s^−1^. Quantum yield of PSII electron transport (Φ_PSII_) was calculated as reported in Maxwell & Johnson (2000). Instantaneous water use efficiency (WUE_i_) was calculated as *A_N_*/*E*. All the measurements were determined on at least six well‐exposed and fully expanded leaves per genotype and treatment.

### Photosynthetic pigment content

The evaluation of total carotenoids and chlorophylls was carried out according to Wellburn (1994) and Zouari *et al*. (2014), as modified by Rigano *et al*. (2016). To obtain the lipophilic extract, 0.30 g sample were extracted with 24 ml acetone/hexane (40/60, v/v). The mixture was centrifuged at 20,000 *g* for 5 min at 4 °C. Supernatants were collected and stored at −20 °C until analyses. For carotenoids and chlorophyll *a* and *b* determination, absorbance of lipophilic extracts was read at 470, 663 and 645 nm, respectively. Results were converted into mg 100 g^−1^ FW. Three separate biological replicates for each sample and three technical assays for each biological repetition were measured.

### Hydrogen peroxide, malondialdehyde, AsA and GSH determination

Quantification of H_2_O_2_ content was carried out using a colorimetric method (Sergiev *et al*. 1997). Briefly, 500 mg frozen leaf powder were extracted with 5 ml ice cold 0.1% trichloroacetic acid (TCA) and the mixture then incubated for 15 min on ice and centrifuged at 9400 *g* for 15 min at 4 °C. To 500 µl supernatant were added 500 µl phosphate buffer 10 mm (pH 7.0) and 1 ml potassium iodide (1 m). The mixtures were then incubated in the dark for 40 min and measured at 525 nm using a Nano photometer (Implen, Munich, Germany). Three separate biological replicates for each sample and three technical assays for each biological repetition were measured. The concentration was expressed in mmol · g^−1^ FW.

The first fully developed leaf was taken for determination of malondialdehyde (MDA). The MDA levels in leaf tissues indicate the levels of membrane lipid peroxidation. Briefly, 0.2 g leaf sample was ground with 1 ml ice cold 0.1% TCA. Samples were incubated for 15 min on ice and centrifuged at 9400 *g* for 10 min at 4 °C. Afterwards, 0.25 ml supernatant was mixed with 1250 ml reaction solution (TCA 20% + thiobarbituric acid [TBA] 0.5%), placed in a water bath for 30 min at 95 °C and measured at 532 and 600 nm with a nano photometer TM (Implen). Three separate biological replicates for each sample and three technical assays for each biological repetition were measured. The concentration was expressed as quantity of MDA‐TBA complex (Zhang & Kirkham 1996).

Quantification of reduced AsA and total AsA (AsA + dehydroascorbate) measurements were carried out using a colorimetric method (Stevens *et al*. 2006) with modifications reported by Francesca *et al*. (2020). Briefly, 500 mg frozen powder from tomato leaves were extracted with 600 µl ice cold 6% TCA and the mixture was then incubated for 15 min on ice and centrifuged at 12,000 *g* for 20 min. For reduced AsA evaluation, to 20 µl supernatant were added to 20 µl 0.4 m phosphate buffer (pH 7.4), 10 µl double distilled (dd) H_2_O and 80 µl colour reagent solution. This solution was prepared by mixing solution A (31% H_3_PO_4_, 4.6% TCA and 0.6% FeCl_3_ [w/v]) with solution B (4% 2,20‐dipyridyl [w/v]). For total AsA, to 20 µl sample, 20 µl 5 mm dithiotreitol in 0.4 m phosphate buffer (pH 7.4) were added and the mixture incubated for 20 min at 37 °C. A total of 10 µl *N*‐ethylmaleimide (NEM; 0.5% [w/v] in water) were added and left for 1 min at room temperature. Then 80 µl colour reagent were added, as previously described for reduced AsA. Both final mixtures were incubated at 37 °C for 40 min and measured at 525 nm using a mano photometer TM (Implen). Three separate biological replicates for each sample and three technical assays for each biological repetition were measured. The concentration was expressed in μmol · g^−1^ FW. For GSH determination, 0.3 g frozen powder from tomato leaves was homogenized with cold 5% metaphosphoric acid at 4 °C in a 1:6 ratio (w/v) in order to obtain deproteinized extracts. After centrifugation at 20,000 *g* for 15 min, the supernatants were collected and used for analysis of GSH content and redox state, according to De Pinto *et al*. (1999). The concentration of reduced and total GSH was expressed in nmol · g^−1^ FW.

### Enzyme antioxidant activity assay

For determination of enzyme activities, 0.5 g frozen powder from tomato leaves was ground to a fine powder in a mortar in the presence of liquid nitrogen and mixed with extraction buffer containing 50 mm Tris‐HCl (pH 7.5), 0.05% cysteine and 0.1% bovine serum albumin (BSA), 1 mm phenylmethylsulfonyl fluoride, 5% polyvinylpolypirrolidone in a 1:4 ratio (w/v). Supernatants obtained after centrifugation at 20,000 *g* for 20 min were used for spectrophotometric analyses.

Cytosolic APX (l‐ascorbate:hydrogen peroxide oxido‐reductase, EC 1.11.1.11) activity was measured by following the H_2_O_2_‐dependent oxidation of AsA at 290 nm in a reaction mixture containing 0.1 m Tris‐acetate buffer, pH 6.4, 350 µm AsA, 170 µm H_2_O_2_, 50 µg protein. CAT (EC 1.11.1.6) activity assay was performed by following H_2_O_2_ dismutation at 240 nm in a reaction mixture consisting of 0.1 m phosphate buffer, pH 7.0, 50 μg protein and 18 mm H_2_O_2_ (ɛ = 39.6 m^−^ ^1^ · cm^−1^). POD (EC 1.11.1.7) activity was measured following the oxidation of 3,3′,5,5′‐tetramelbenzidine at 652 nm (ɛ = 26.9 mm^−^ ^1^ · cm ^−^ ^1^).

Protein content was determined according to Bradford (1976) using BSA as standard. All enzyme activities were measured using a Beckman (Fullerton – CA) DU 7000 spectrophotometer.

For all analyses, three separate biological replicates for each sample and three technical assays for each biological repetition were measured.

### Analysis of RRS data

In order to detect hypothetical private loci in E42 and LA3120, available RRS data on 27 genotypes, including LA3120 and E42, from a previous experiment (Zouari *et al*. 2014) were retrieved. The 27 tomato genotypes were selected from a wide tomato germplasm collection available at the University of Naples Federico II (Table S1; Sacco *et al*. 2015) and are hosted on the LabArchive repository (http://dx.doi.org/10.6070/H4TT4NXN). Genomic DNA was extracted from 100 mg young leaf tissue using the DNeasy plant mini kit (Qiagen). DNA concentration was determined using a Qubit fluorometer (Invitrogen, Carlsbad, CA, USA). The 260/280 and 260/230 nm ratios were estimated with a NanoDrop spectrophotometer (Thermo‐Fischer Scientific, Waltham, MA, USA). DNA sequencing was performed using 1 µg DNA diluted in 30 µl sterile Milli‐Q water to constitute libraries for the double digest restriction‐site associated DNA sequencing (ddRAD‐seq), as reported by Peterson *et al*. (2012). The double digestion reaction was performed using the restriction enzymes *Mbo*I and *Sph*I, and the fragments were sequenced using the V4 chemistry paired end 125 bp mode on a HiSeq2500 instrument (Illumina, San Diego, CA, USA). The demultiplexing step was performed using Stacks version 2.0. The high‐quality reads were aligned to the reference genome of *S*. *lycopersicum* (version SL4.0) using BWA‐MEM with default parameters through the software Samtools 1.6 (Li & Durbin 2009), by selecting reads mapping uniquely on the genome. The raw variants were filtered and manipulated using VCFtools version 0.1.13 (http://vcftools.sourceforge.net) by setting the following parameters: minimum mean of Depth of Coverage (min‐mean DP) = 5, maximum missing data (max‐missing) = 0.5. Heterozygous loci were manually removed. The annotation and prediction of the possible effect of the SNPs were evaluated using SnpEff (http://snpeff.sourceforge.net/) (Cingolani *et al*. 2012) and iTAG4.1 tomato annotation version as reference.

### Statistical analyses

Data were subjected to two‐way analysis of variance (anova). To separate means within each parameter, the Tukey’s test was performed. Differences at *P* < 0.05 were considered to be significant. anova and principal components analysis (PCA) were performed by using SPSS (Statistical Package for Social Sciences) Package 6, version 23.0.

## RESULTS

### Effect of single and combined abiotic stresses on plant growth

**Fig. 1:**
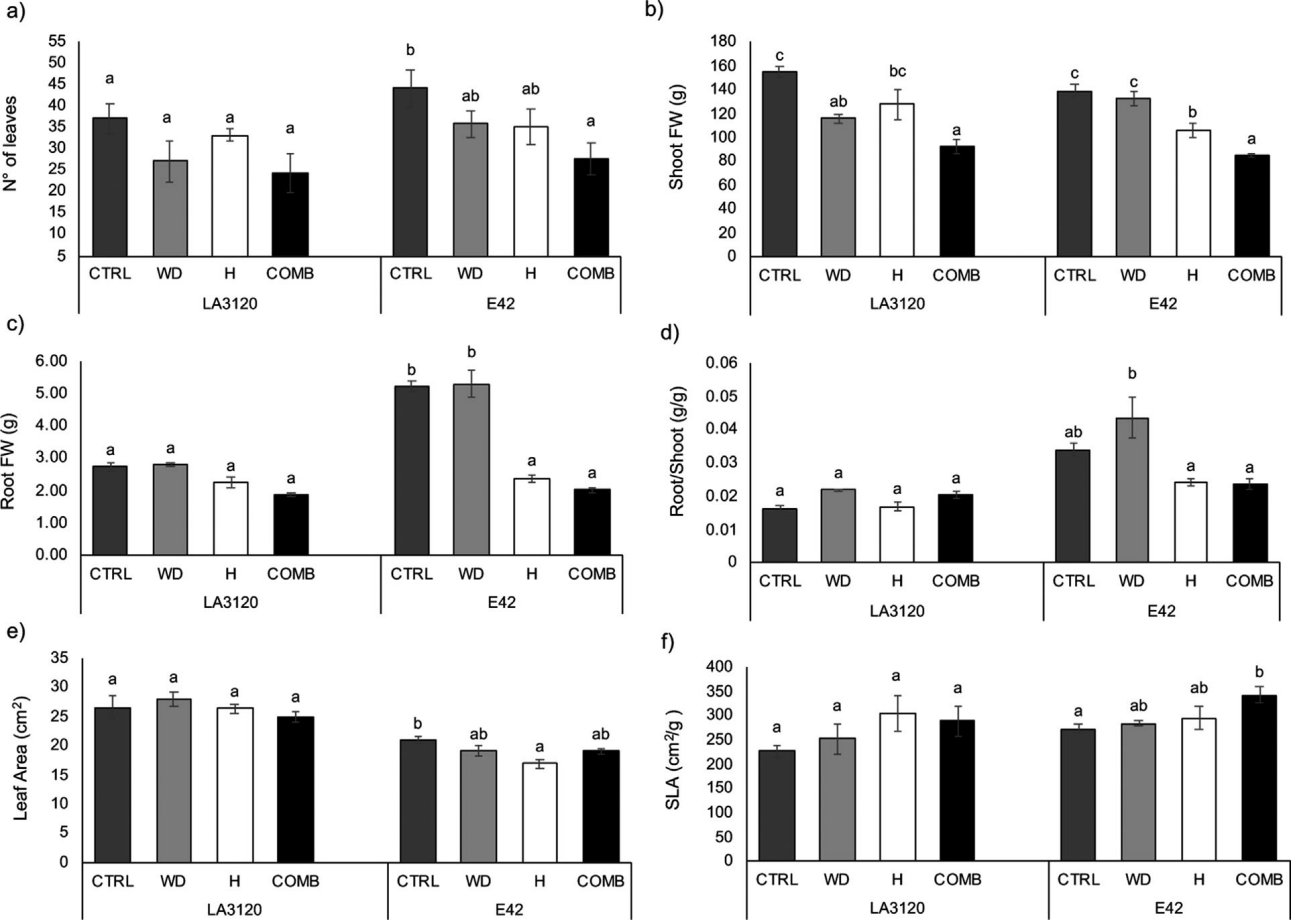
Plant growth parameters of two tomato genotypes under control (CTRL), water deficit (WD), heat (H) and combined stresses (COMB). (a) Leaf number, (b) shoot fresh weight, (c) root fresh weight, (d) root/shoot ratio, (e) leaf area and (f) specific leaf area (SLA). The data are mean ± standard error (SE) of three replicates for leaf number, shoot fresh weight, root fresh weight and root/shoot ratio. Data are mean ± SE of five leaves per genotype per treatment for leaf area and SLA. Within each tomato line, different letters indicate significant differences among treatments (*P* < 0.05).

The effect of single and combined stresses was investigated in plants (30‐day‐old) stressed for a 3‐week period. Water deficit, temperature and combined stresses were not statistically significant for leaf number in LA3120 compared to the non‐stressed control, although there was a tendency to decrease (Fig. 1a). In contrast, the combined stresses had a significant effect on leaf number in E42, which was lower in response to this treatment compared to the control (Fig. 1a). Shoot FW of LA3120 decreased under limited water availability and combined stresses compared to the control; in contrast, shoot FW of E42 decreased under both heat and combined stresses (Fig. 1b). Root FW of E42 was affected by heat and combined treatments but was not statistically different in LA3120 compared to the control, although there was a tendency to decrease (Fig. 1c). The root/shoot ratio did not change in LA3120 in response to different treatments, whereas in E42 there was a tendency to increase under water‐limiting condition and decrease under heat and combined stresses (Fig. 1d). LA was significantly lower under heat treatment only in the genotype E42 compared to the control (Fig. 1e). Moreover, the SLA of E42 increased under combined stresses compared to control plants, while in LA3120 there was no change in response to the different treatments (Fig. 1f). In both genotypes, plants under heat and combined stress showed a strong reduction in pollen viability compared to the control treatment. In particular, in genotype LA3120, heat treatment decreased pollen viability by 61.61%, while in E42 and combined treatments pollen viability decreased by 94.63% (Table 1). In E42, the fruit FW also decreased under water stress (Table 1). Consistent with the pollen viability data, no fruits were present on plants of either genotype subjected to heat and combined stresses (Table 1).

**Table 1:** Pollen viability and fresh weight of fruits of two tomato genotypes under control (CTRL), water deficit (WD), heat (H) and combined stresses (COMB).

genotype	treatment	pollen (%)	fruit FW (g)	no. of fruits
LA3120	CTRL	80.82 ± 0.05 b	95.73 ± 5.14 b	6.75 ± 2.06 b
	WD	80.58 ± 0.07 b	86.29 ± 5.20 b	5.6 ± 3.50 b
	H	31.02 ± 0.20 a	0 ± 0.00 a	0 ± 0.00 a
	COMB	22.22 ± 0.25 a	0 ± 0.00 a	0 ± 0.00 a
E42	CTRL	79.63 ± 0.06 c	78.85 ± 3.43 c	9.25 ± 2.87 b
	WD	73.11 ± 0.04 c	37.06 ± 14.09 b	11 ± 4.69 b
	H	33.77 ± 0.20 b	0 ± 0.00 a	0 ± 0.00 a
	COMB	4.28 ± 0.01 a	0 ± 0.00 a	0 ± 0.00 a

FW = fresh weight; SE = standard error.

Data are mean ± SE of three replicates. Within each tomato line, different letters indicate, for each variable (pollen, fruit FW and No. of fruits), significant differences among treatments (*P* < 0.05).

### Effects of single and combined stresses on leaf gas exchange and fluorescence

**Fig. 2:**
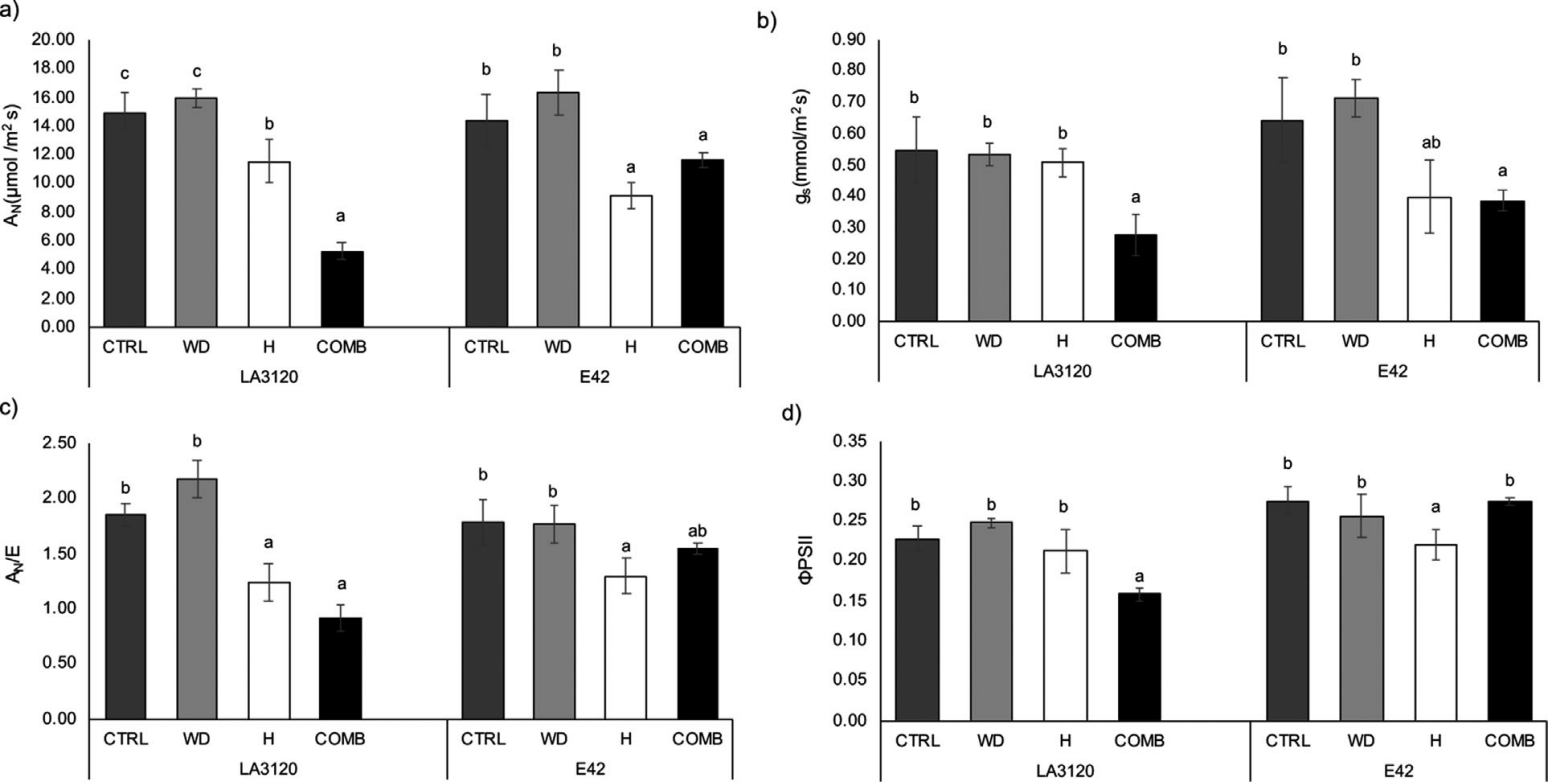
Gas exchange and fluorescence measurements in leaves of two tomato genotypes under control (CTRL), water deficit (WD), heat (H) and combined stresses (COMB). (a) Net photosynthesis (*A_N_*), (b) stomatal conductance (*g_s_*), (c) water use efficiency (*A_N_*/*E*), (d) quantum yield of PSII (Φ_PSII_). Data are mean ± standard error (SE) of six leaves per genotype per treatment. Within each tomato line, different letters indicate significant differences among treatments (*P* < 0.05).

The imposed stresses negatively affected leaf gas exchange in both genotypes (Fig. 2a–c). Net photosynthesis (*A_N_*) and *g_s_* were more affected when heat and water deficit were simultaneously applied than when applied as single stresses (Fig. 2a and b). In particular, under combined stresses, genotype LA3120 showed a decrease in net photosynthesis of 64.75% compared to the control, while genotype E42 had a 18.99% reduction for this parameter. In LA3120 water use efficiency (*A_N_*/*E*) significantly decreased both under heat and combined stresses (−32.95% and −50.80%, respectively) (Fig. 2c), whereas in E42 it was significantly reduced only under heat stress (−27.71%) compared to the control. The quantum yield of PSII (Φ_PSII_) was negatively affected in LA3120 genotype only under combined stresses conditions, whereas in E42 it was reduced only under heat conditions (Fig. 2d).

### Effects of single and combined stresses on pigment and antioxidant content

**Fig. 3:**
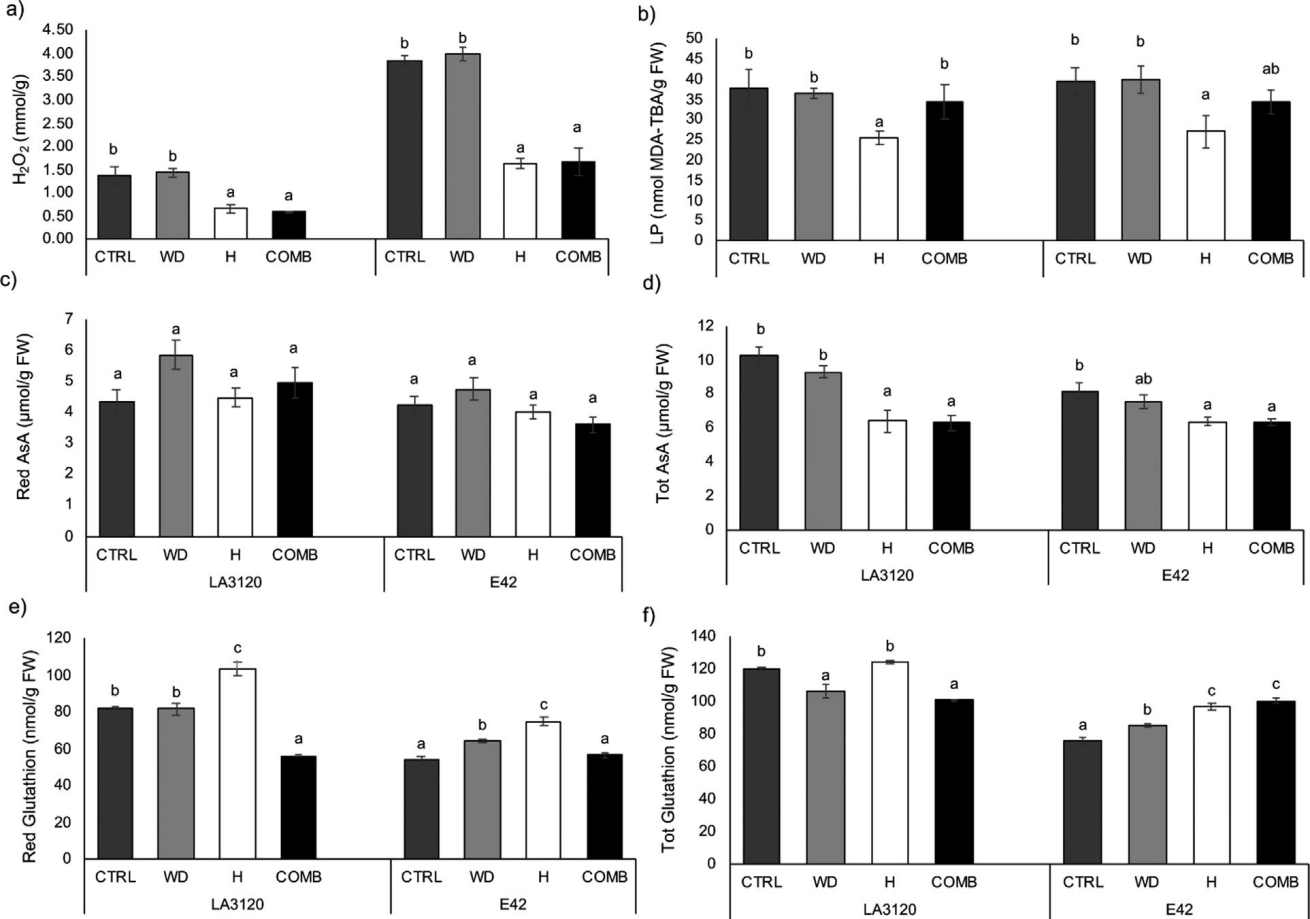
Oxidative stress markers and hydrophilic antioxidants of two tomato genotypes under control (CTRL), water deficit (WD), heat (H) and combined stress (COMB). (a) Hydrogen peroxide (H_2_O_2_), (b) lipid peroxidation (LP), measured as malondialdehyde‐thiobarbituric acid (MDA‐TBA) content, (c) reduced ascorbic acid (AsA), (d) total AsA, (e) reduced and (f) total glutathione content. Data are mean ± standard error (SE) of three replicates. Within each tomato line, different letters indicate significant differences among treatments (*P* < 0.05).

High temperatures and combined stresses increased the content of carotenoids, and chlorophylls *a* and *b* in both genotypes (Table 2). Specifically, under elevated temperatures LA3120 genotype had increases of 109.54%, 85.21% and 145.60% in total carotenoids, chlorophyll *a* and chlorophyll *b*, respectively, compared to the control. In LA3120, the highest content of photosynthetic pigments compared to control was under heat stress conditions, followed by the combined stress treatment. In E42, the combined stresses increased carotenoid content 47.07% and chlorophylls *a* and *b* by 38.50% and 61.07%, respectively, compared to the control. Conversely, under limited water availability chlorophyll *a* significantly decreased in this genotype. To verify if stress conditions were responsible for oxidative damage in the two genotypes, the concentrations of H_2_O_2_ and MDA were determined (Fig. 3). Surprisingly, H_2_O_2_ content of LA3120 and E42 significantly decreased under heat and combined stress compared to the control. The highest concentration of H_2_O_2_ was in plants grown in water‐limiting conditions at 1.44 mmol · g^−1^ in LA3120 and 3.97 mmol · g^−1^ in E42 (Fig. 3a). Similarly, MDA content in both genotypes significantly declined under high temperatures compared to the control treatment (Fig. 3b). The different stress conditions did not change the content of reduced AsA (Fig. 3c). However, total AsA decreased under heat and combined treatments in both genotypes. Specifically, in genotype LA3120 there was a 36.62% lower content of total AsA under combined stress (Fig. 3d). In both genotypes, reduced GSH content increased under heat stress and decreased with the combined stress (Fig. 3e). In genotype LA3120, there was a decrease in the total GSH pool under water deficit and combined stress. Conversely, the content of total GSH significantly increased in E42 subjected to all stress conditions (Fig. 3f). However, a significant reduction in the GSH redox state was observed in both genotypes subjected to combined treatment.

**Table 2:** Pigments content in two tomato genotypes under control (CTRL), water deficit (WD), heat (H) and combined stress (COMB).

genotype	treatment	total carotenoids (mg 100 g^−1^ FW)	chlorophyll *a* (mg 100 g^−1^ FW)	chlorophyll *b* (mg 100 g^−1^ FW)
LA3120	CTRL	26.58 ± 3.31 a	78.18 ± 4.67 a	30.42 ± 4.24 a
	WD	27.11 ± 2.61 a	76.41 ± 5.78 a	33.15 ± 5.37 a
	H	55.69 ± 5.73 c	144.80 ± 14.78 c	74.71 ± 3.03 c
	COMB	42.97 ± 4.82 b	107.64 ± 8.50 b	50.95 ± 6.94 b
E42	CTRL	32.99 ± 4.31 a	95.76 ± 5.06 b	37.15 ± 4.22 a
	WD	40.67 ± 7.44 b	82.71 ± 8.42 a	56.38 ± 7.75 b
	H	43.48 ± 5.16 bc	106.70 ± 3.08 c	52.86 ± 6.39 b
	COMB	48.52 ± 3.41 c	132.63 ± 2.97 d	59.84 ± 5.16 b

SE = standard error.

Data are mean ± SE of three replicates. Within each tomato line, different letters indicate, for each variable (total carotenoids, chlorophyll *a* and chlorophyll *b*), significant differences among treatments (*P* < 0.05).

### Effects of single and combined stresses on antioxidant enzyme activity

**Fig. 4:**
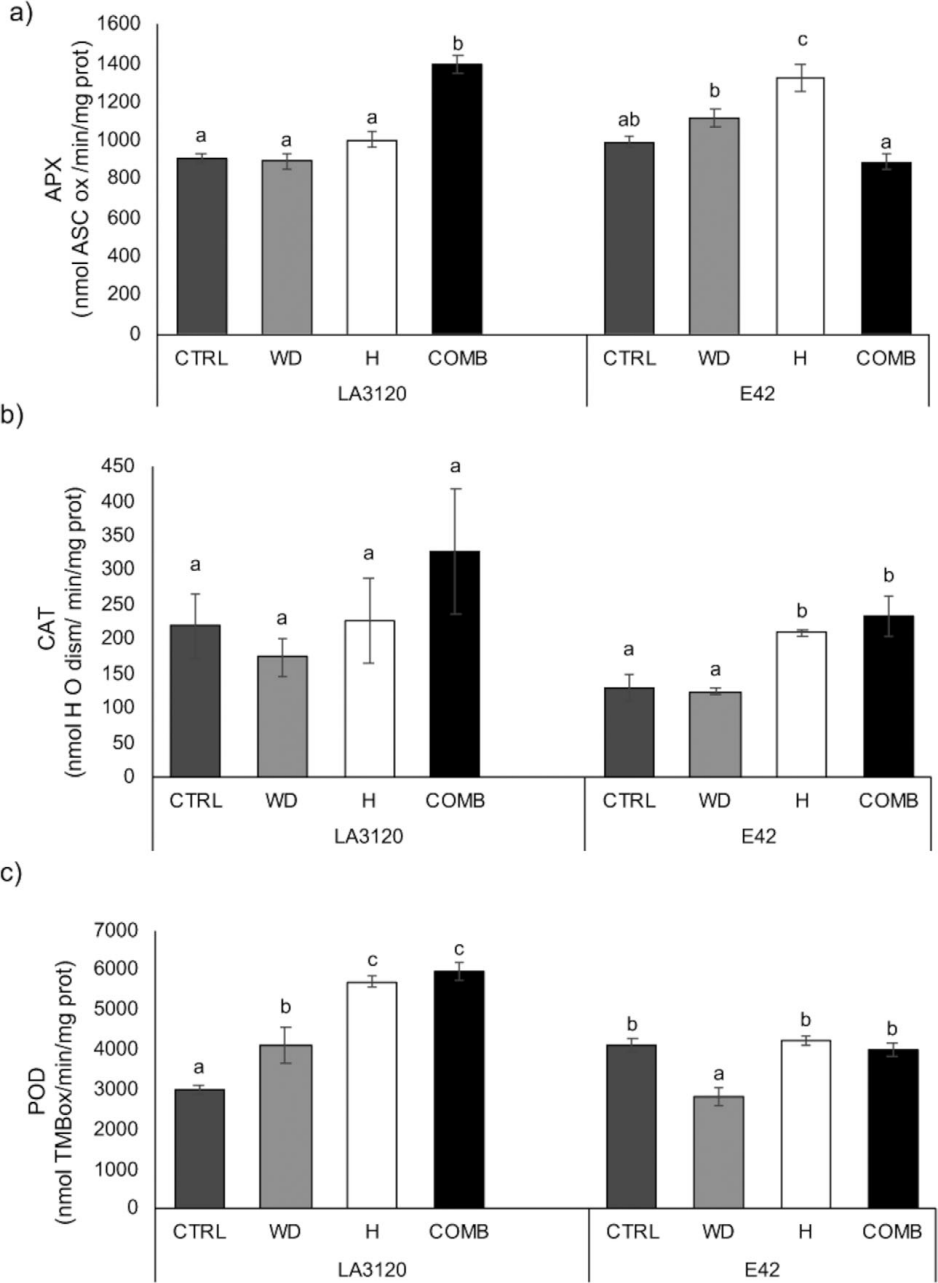
Activity of reactive oxygen species (ROS) removal enzymes in two tomato genotypes under control (CTRL), water deficit (WD), heat (H) and combined stress (COMB). (a) Ascorbate peroxidase (APX), (b) Catalase (CAT), (c) Peroxidases (POD). Data are mean ± standard error (SE) of three replicates. Within each tomato line, different letters indicate significant differences among treatments (*P* < 0.05).

The enzymes involved in ROS scavenging responded very differently in the two genotypes. In LA3120, APX was not significantly affected by water deficit and heat given individually; however, the combined treatment significantly increased APX activity. On the other hand, in E42 only heat stress was able to induce APX activity, which remained comparable to the control under water deficit and combined stress (Fig. 4a). The activity of CAT after water deficit, heat and combined stress treatment did not significantly change in LA3120. Conversely, an increase in CAT occurred after heat and combined stress in the E42 genotype (Fig. 4b). In LA3120 subjected to all stress conditions an increase in POD activity was observed, even if the extent of the increase was higher after heat and combined treatment. In E42, POD activity did not change after heat and combined stress and significantly decreased in plants subjected to water deficit (Fig. 4c).

### Principal components analysis

**Fig. 5:**
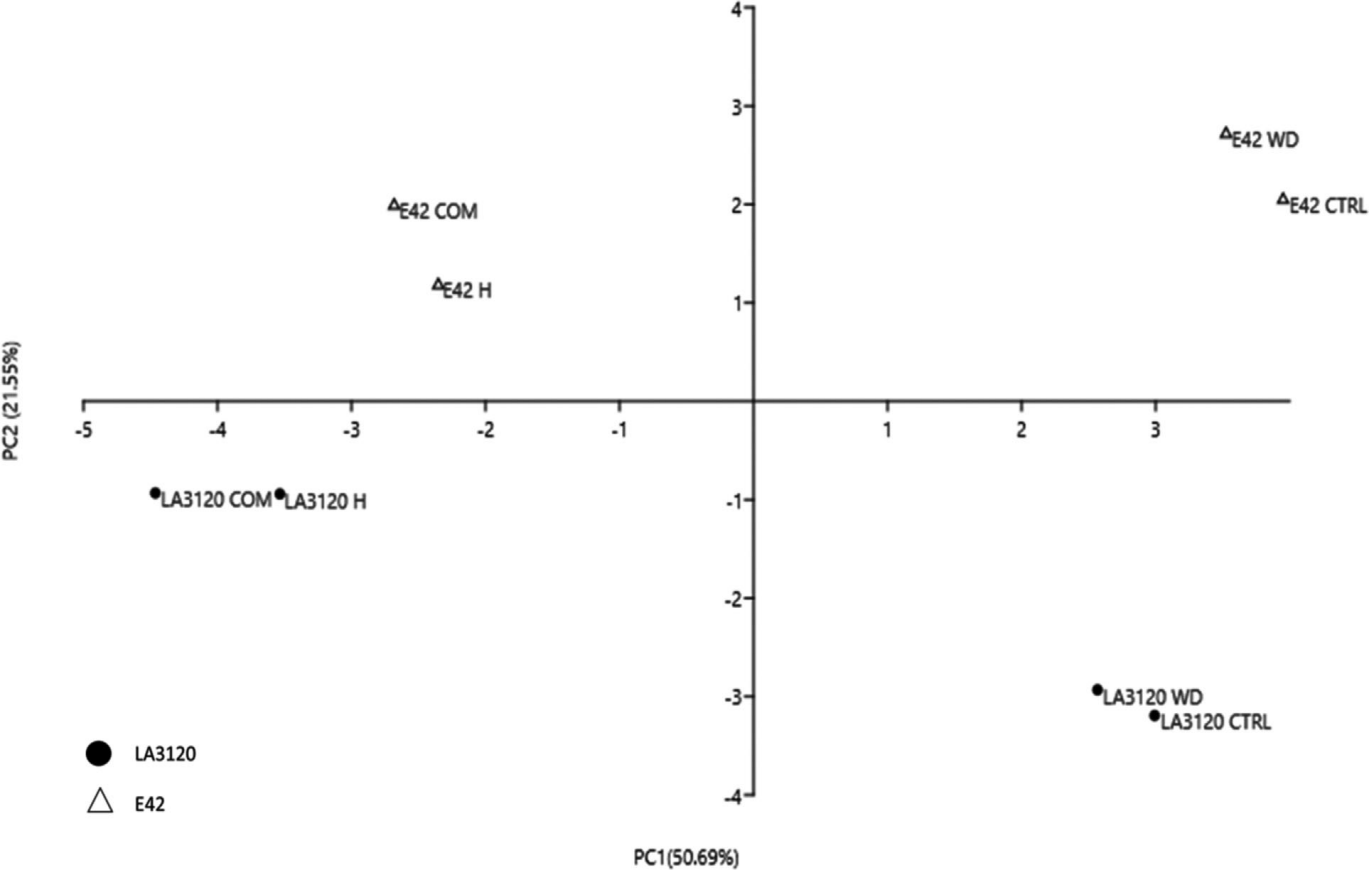
Principal component loading plot and scores of principal components analysis (PCA) in two tomato genotypes under control (CTRL), water deficit (WD), heat (H) and combined (COMB) stress.

To provide a broad overview of the different analyses conducted on the two tomato genotypes in response to the different stresses applied, a PCA was conducted. Based on our experimental data, seven principal components (PCs) were associated with Eigenvalues >1 and accounted for 100% of the total variance, with PC1, PC2, PC3, PC4, PC5, PC6 and PC7 accounting for 50.69%, 21.55%, 10.98%, 6.96%, 5.12%, 2.71% and 1.98%, respectively (Table S2). PC1 was the primary driver for differences between genotypes (Fig. 5) and the main parameters leading to this separation were fruit FW, pollen viability, *g_s_*, fruit number and *A_N_* (Table S2). There was also a treatment‐dependent clustering, with the primary differences driven by PC2 (Fig. 5). The main parameters of PC2 were SLA, root/shoot ratio and H_2_O_2_ content (Table S2).

### RRS analysis

In order to identify unique SNP variants in genes related to the stress response in the two genotypes, RRS data deriving from a previous investigation were here analysed (Table S1) (Olivieri *et al*. 2020). The SNP resulted in 108,936 different variants, which were reduced to 17,283 after filtering. These consisted of 16,328 SNP and 955 InDel variants. Among them, 251 were hypothetical private for LA3120 and 6086 for E42, corresponding to ˜37% of the total filtered SNP dataset (Table 3). The snpEff analysis was carried out to identify variants with a significant impact on the protein function and resulted in 54 SNP with moderate impact (of which 53 were private for E42) and three SNP with high impact (all private for E42). As a whole, the variants affected 1,398 genes, of which 47 harboured variants with high and moderate impacts (Table S3 **)**. Based on their annotation, we identified a subset of genes potentially involved in abiotic stress response: *Solyc01g056890* coding for the curvature thylakoid protein; *Solyc07g021200* coding for the large chain subunit of RUBISCO; *Solyc01g079380* coding for a GRAS transcription factor; *Solyc07g053640* coding for the arabinogalactan (AGP) protein; and *Solyc02g086610* coding for an isocitrate dehydrogenase. All these genes are characterized by a high impact private variant in genotype E42.

**Table 3:** Summary of the private loci detected in E42 and LA3120 tomato lines.

genotype	loci (no.)	high	moderate	low	modifier	affected genes
E42	6086	3	53	34	5996	1268
LA3120	251	0	1	1	249	130
Total	6337	3	54	35	6245	1398

The number of analysed loci, snpEff impact and number of affected genes are also reported.

## DISCUSSION

Water stress and high temperatures, alone or in combination, can greatly affect world crop production. Previous studies have clearly shown that the responses of crops to each individual stress do not necessarily reflect plant responses to combined abiotic stresses, a much more frequent condition in nature and agricultural contexts. In order to study the different mechanisms engaged by tomato plants to withstand combined abiotic stresses, two putative heat stress‐tolerant genotypes were selected to test whether their genetic/physiological traits were also useful to overcome water shortage and combined stresses (Moles *et al*. 2018). LA3120 is a heat‐tolerant *S. lycopersicum* accession retrieved from the Tomato Genetic Resources Center, whereas E42 was selected in our laboratory as a high and stable yielding genotype when grown under high temperatures in the open field (Olivieri *et al*. 2020). For these genotypes, physiological responses to both limited water availability and elevated temperatures in response to long‐term stress were analysed, highlighting clear differences, and also similarities, in the two genotypes.

Considering all the analysed traits, heat stress had a predominant effect over limited water availability on both tomato genotypes subjected to combined stress. This was also evident from the PCA (Fig. 5) that clearly separated samples subjected to heat and combined stress from control samples and samples subjected to water stress. Indeed, considering the leaf gas exchange measurements and oxidative markers, both genotypes were not significantly affected by water shortage, thus showing high ability to overcome long periods with reduced water availability. Interestingly, however, E42 had a higher tolerance to moderate and prolonged water stress compared with LA3120. Indeed, E42 was able to preserve the same shoot and root FW under limited water availability, proving its ability to maintain high plant carbon gain. Moreover, in E42, a higher root/shoot ratio was recorded under water stress, a specific trait that has been previously reported for drought‐tolerant genotypes (Moles *et al*. 2018), and which may have provided an advantage in terms of nutrient and water uptake in E42.

Considering heat stress, although both genotypes were previously classified as heat stress‐tolerant lines under field conditions (Arena *et al*. 2020), severe effects on plant performance were observed in both genotypes in response to a 3‐week stress. That said, LA3120 could adapt better to chronic heat stress, as indicated by the significant decreases in shoot and root FW recorded only in E42 in response to heat treatment. Under heat stress, a decrease in LA was also observed in E42, which may indicate a tendency to reduce water loss by transpiration. This could be a specific adaptive mechanism to maintain higher leaf water content in response to high temperature stress.

One of the phases most sensitive to heat stress is the reproductive phase. Exposure to high temperatures is known to affect pollen viability, fertilization and fruit formation (Balfagón *et al*. 2018). Accordingly, prolonged exposure to high temperatures compromised pollen viability and no fruits were formed in either genotype under heat stress. In both genotypes, heat stress impaired CO_2_ fixation and reduced the instantaneous *A_N_*/*E*. This is indicative of biochemical limitations of photosynthesis, including Rubisco activity (Vitale *et al*. 2008). However, unlike E42, where electron transport was downregulated, heat stress alone did not lead to a reduction in PSII efficiency (ΦPSII) in LA3120.

Both LA3120 and E42 were able to activate effective antioxidant defence mechanisms in response to heat stress. The accumulation of antioxidant compounds contributes to prevent oxidative damage and lipid peroxidation and, thereby, protects cell membrane integrity (Zhou *et al*. 2019, 2020a). It has been previously shown that in wheat genotypes exposed to heat stress, low levels of membrane damage are positively correlated with chlorophyll and antioxidant content (Almeselmani *et al*. 2006; Hameed *et al*. 2012). Accordingly, in both tomato genotypes used in this study, heat stress increased the content of chlorophylls and carotenoids, as already shown in other heat‐tolerant tomato genotypes (Zhou *et al*. 2015). Moreover, accumulation of GSH occurs in both genotypes under heat stress. Interestingly, accumulation of H_2_O_2_ and MDA under heat stress decreases in both genotypes, and these could be considered as markers of stress tolerance, as previously demonstrated (de Pinto *et al*. 2015; Zhou *et al*. 2019). Many studies on sensitive and thermotolerant genotypes of the same species highlight a strong relationship between thermotolerance and the capacity to increase one or more ROS scavenging enzymes. For instance, a study conducted in different wheat genotypes found that heat tolerance is associated with the ability of APX and CAT to cooperate in the removal of H_2_O_2_ (Dash & Mohanty 2002). In particular, APX plays a key role in the scavenging of H_2_O_2_ in heat stress response (de Pinto *et al*. 2015; Zhou *et al*. 2020b). Therefore, the activation of APX and CAT under heat stress in E42 may be partly responsible for the lower levels of H_2_O_2_ observed. The enzyme POD also plays an important role in decreasing H_2_O_2_ content, contrasting membrane lipid peroxidation and maintaining cell membrane integrity (Jaleel *et al*. 2008), and its activity is related to the water retention of leaves (Zhou *et al*. 2020b). Thus, activation of POD evidenced in LA3120 under elevated temperatures may play a key role in the response mechanisms activated in this heat‐tolerant genotype.

Prolonged elevated temperatures had a predominant effect over limited water availability in both genotypes, although, in line with previous results (Zhou *et al*. 2017b), combined stresses caused more severe damages to plant growth parameters than individual stresses. Indeed, a drop in leaf number and in shoot and root FW was only observed under combined stress. The prolonged combined stress compromised viability of pollen and, as already seen under heat stress alone, no fruits were formed in either genotype under combined heat and water stress. More dramatic effects were, however, evidenced in E42, where pollen viability dramatically dropped under combined stress compared with heat stress alone. Based on the monitored growth parameters, it can be hypothesized that E42 and LA3120 used different strategies to overcome the combined stresses. Conversely, the sensitivity to combined stress varied significantly between the two genotypes. Indeed, in LA3120 the combined stress induced a strong decline in *A_N_*, followed by a decrease in *g_s_* and quantum yield of PSII electron transport, suggesting that the combined stress determined both stomatal and non‐stomatal limitation to carbon assimilation. On the contrary, in E42 plants subjected to combined stress, photosynthesis was preserved, and the electron transport rate increased compared with plants subjected to heat stress. Under combined stress, that limited CO_2_ assimilation, photosynthetic electron flow departed more towards pathways other than CO_2_ fixation, so maintaining oxidized the electron transport chain and preventing irreversible oxidative damage. Based on our data on antioxidant enzyme activity, gas exchange and florescence measurements, we hypothesized a more important role of photorespiration in E42 than in LA3120 in sustaining electron transport under combined stress, as indicated by the observed increase in CAT activity. In contrast, occurrence of the AsA‐GSH cycle would seem the main alternative pathway to CO_2_ assimilation under combined stress in LA3120 (Vitale *et al*. 2020). Under combined stress, water use efficiency dropped only in LA3120, whereas E42 showed a better capacity to use limiting water resources in this condition. Analyses of functional leaf traits under combined stress in the two genotypes seem to confirm this hypothesis. Indeed, E42 had higher SLA values compared with non‐stressed samples, indicating a better state of leaf hydration and a higher photosynthetic capacity per unit leaf dry biomass. The increased SLA under combined stress may represent a further adaptive strategy of leaf morphological traits to the changing environment in order to maximize the photosynthesis (Vitale *et al*. 2020), by investing less dry matter in protective tissues (lower leaf construction cost) and more nitrogen to photosynthesis (higher chlorophyll content). As for the leaf shape, both genotypes have a regular leaf shape, with serrated leaf edges. The colour of the leaves varies, from light green in the LA3120 genotype to intense dark green for E42. Both genotypes had some severely curved leaves with yellow spots at the end of the heat and combined stress treatments. Considering the antioxidant defence responses, the adaptive strategies activated in response to the combined stress were similar to those activated for heat stress alone, and a decrease in accumulation of H_2_O_2_ and an increase in chlorophyll and carotenoid content in both genotypes was observed also in these conditions. However, accumulation of total GSH occurred only in E42 under combined stress. The activation of APX under combined stress in LA3120 may be partly responsible for the lower levels of H_2_O_2_ observed. Similar results were previously observed in tomato subjected to heat stress and drought (Zhou *et al*. 2019). The increase in CAT activity in E42 subjected to combined stress, in the absence of APX activation, could be a compensatory mechanism of this genotype to maintain low levels of MDA and oxidative stress (Gill & Tuteja 2010). On the other hand, the tolerance to combined stress of LA3120 may be related to the simultaneous induction of APX and POD activity. In particular, the activation of POD evidenced in LA3120 under all the studied stress conditions may play a key role in the defence mechanisms activated by this heat‐tolerant genotype.

In a previous work (Olivieri *et al*. 2020), RRS analysis performed on a group of genotypes allowed us to demonstrate that E42 has distinct genetic diversity compared with the other genotypes analysed, probably related to a particular breeding history that likely included crossing events with wild tomato species, including *S. pimpinellifolium* (Olivieri *et al*. 2020). Herein, we retrieved RRS data from 27 genotypes and analysed their nucleotide variability. The analysis demonstrated that E42 harbours 56 hypothetical private mutations within the coding regions of 46 genes that can have a significant impact on protein functions, whilst only one private allele with moderate impact was evidenced in LA3120 compared with the other genotypes. Among the hypothetical private variants affecting E42, one mutation is in the *Solyc01g056890* gene, which codes for a curvature thylakoid protein, known to be involved in the responses to different light intensities. In particular, Trotta *et al*. (2019) demonstrated that the dynamics of thylakoid protein complexes are crucial in the optimization of photosynthesis under fluctuating light intensities (Trotta *et al*. 2019). A mutation was also detected in the *Solyc07g021200* gene coding for the large chain subunit of the ribulose‐1,5‐bisphosphate carboxylase. It has been reported that under high temperatures, Rubisco activation state decreases, an event correlated with changes in the rate of electron transport (Perdomo *et al*. 2017). This is particularly interesting considering that in our study E42 was able to preserve photosynthesis and increase electron transport rate when subjected to combined heat and water stress. During heat treatments in tomato, a small number of Rubisco isoforms, probably the more stable and efficient ones, are used (Parrotta *et al*. 2020). Therefore, in future, additional analyses should be conducted on the candidate gene coding for the isoform here detected in order to obtain further insights into its involvement in heat stress responses. The other two gene variants scored in this study, that were already identified in our previous analyses, mapped within the transcription factor GRAS (*Solyc01g079380*) and the isocitrate dehydrogenase (*Solyc02g086610*), both involved in plant development and responses to abiotic stresses (Olivieri *et al*. 2020). A mutation was detected in the *Solyc07g053640* gene coding for the AGP protein. AGPs in leaves are involved in the increase in cell wall thickness and stiffness, thus limiting water loss and maintaining turgor pressure and cell integrity under elevated temperatures and drought (Mareri *et al*. 2019). In particular, this mutation could explain the capacity of E42 to use limiting water resources when exposed to elevated temperatures, the better state of leaf hydration and the higher photosynthetic capacity per unit of leaf dry biomass of this genotype when subjected to combined abiotic stresses. Finally, two variants with high impact, but on genes of unknown function, were also found that are of interest and should be further investigated.

## CONCLUSION

Novel tomato genetic resources can carry valuable traits for adaptation to stressful environments, such as water stress and high temperatures. In this study, one novel tomato genotype resistant to high temperatures and one known heat‐tolerant genotype were used in order to analyse the different strategies developed in response to single and combined abiotic stresses (high temperature and water shortage). Noteworthy, both lines seemed to be tolerant to the prolonged water shortage applied in our experiment. Heat and combined abiotic stresses instead clearly distinguished the two genotypes, which employed different physiological responses in order to counteract the applied stresses. That said, both genotypes were able to employ efficient antioxidant defence mechanisms in response to single and combined stress, which could be a key trait for the tolerance observed in both genotypes also in open fields. The identification of candidate genes, obtained by combining the phenotypic and genotypic analyses carried out in this work, might help to dissect this complex trait and could explain the different physiological response to stress observed in the novel genotype E42 compared with LA3120. In particular, this analysis allowed us to confirm the high genetic variability of this novel genotype and detect mutations in candidate genes that should be further analysed, including one in a gene coding for an AGP protein. In conclusion, this paper highlighted the presence of interesting stress tolerance traits, both in a heat‐tolerant genotype (LA3120) and in a novel genotype (E42) selected from an arid and warm region of southern Italy, which should be further studied and could be used in future breeding programmes in order to improve tolerance to abiotic stress in commercial varieties.

## AUTHOR CONTRIBUTIONS

S.F.: conceptualization, formal analysis, investigation, methodology, writing original draft, review and editing; L.V. and C.A.: formal analysis, investigation, writing original draft, review and editing; G.R. and A.P.: formal analysis, investigation; V.C.: writing, review and editing; M.C.d.P. and F.O.: formal analysis, investigation, writing, review and editing; A.M. and A.B.: funding acquisition, supervision, writing, review and editing; M.M.R.: conceptualization, formal analysis, investigation, methodology, supervision, writing original draft, review and editing.

## CONFLICTS OF INTEREST

The authors have no conflicts of interest to declare.

## Supporting information


**Table S1**. List of 27 genotypes sequenced using RRS strategies. The common names and their origin are also reported.

**Table S2**. Eigenvalues, relative and cumulative percentage of total variance, and correlation coefficients for each character.

**Table S3**. List of the 57 variants with a moderate and high impact effect on the protein structure of 47 genes.

## Data Availability

All data generated or analysed during this study are included in this published article [and its supplementary information files].
