# Supplementary material for: The efficient physiological strategy of a novel tomato genotype to adapt to chronic combined water and heat stress
Source: Plant Biol (Stuttg). 2021 Oct 4;24(1):62–74. doi: 10.1111/plb.13339 (PMC9293464; doi:10.1111/plb.13339)
Supplement: Supplementary file 1 — Table S1. List of 27 genotypes sequenced using RRS strategies. The common names and their origin are also reported. [file PLB-24-62-s002.docx]

**Table S1.** List of 27 genotypes sequenced using RSS strategies. The common name and their origin are also reported.

| Genotype | Common name | Origin |
| --- | --- | --- |
| E7 | Corbarino PC04 | Italy |
| E8 | Corbarino PC05 | Italy |
| E17 | Pantano Romanesco | Italy |
| E20 | Pizzutello | Italy |
| E23 | SanMarzano 1-38 SMEC | Italy |
| E30 | Sel PC07 | Italy |
| E36 | Seccagno | Italy |
| E37 | Vesuvio | Italy |
| E40 | Giagiù | Italy |
| E41 | Parmitanella | Italy |
| E42 | PI15250 | Italy |
| E43 | Principe Borghese | Italy |
| E45 | SM246 | Italy |
| E48 | Vesuvio 2001 | Italy |
| E53 | LA0147 | Honduras |
| E55 | LA0358 | Colombia |
| E75 | Gold Nugget | America |
| E76 | Black Plum | America |
| E107 | E-L-19 | Spain |
| E201 | Siccagno | Italy |
| PDVIT | Cannellino Vitiello | Italy |
| LA2662 | Saladette |  |
| LA3120 | Malintka 101 |  |
| DOCET | DOCET | Italy |
| JAG8810 | JAG8810 | Italy |
| M82 | M82 |  |
| E72 | Money Maker | America |
