# Supplementary material for: The efficient physiological strategy of a novel tomato genotype to adapt to chronic combined water and heat stress
Source: Plant Biol (Stuttg). 2021 Oct 4;24(1):62–74. doi: 10.1111/plb.13339 (PMC9293464; doi:10.1111/plb.13339)
Supplement: Supplementary file 2 — Table S2. Eigenvalues, relative and cumulative percentage of total variance, and correlation coefficients for each character. [file PLB-24-62-s001.docx]

**Table S2.** Eigenvalues, relative and cumulative percentage of total variance, and correlation coefficients for each character.

| **Principal Components** | **PC1** | **PC2** | **PC3** | **PC4** | **PC5** | **PC6** | **PC7** |
| --- | --- | --- | --- | --- | --- | --- | --- |
| Eigen value | 12.67 | 5.39 | 2.75 | 1.74 | 1.28 | 0.68 | 0.49 |
| Relative variance (%) | 50.69 | 21.55 | 10.98 | 6.96 | 5.12 | 2.71 | 1.98 |
| Cumulative variance (%) | 50.69 | 72.24 | 83.23 | 90.19 | 95.31 | 98.02 | 100.00 |
| *Eigen vectors* |  |  |  |  |  |  |  |
| Fruit FW | **0.247** | -0.181 | -0.031 | -0.070 | -0.088 | 0.165 | -0.133 |
| Pollen viability | **0.261** | -0.112 | -0.026 | 0.184 | 0.024 | -0.022 | -0.116 |
| Stomatal conductance (gs) | **0.242** | 0.089 | 0.207 | 0.193 | 0.147 | 0.041 | 0.116 |
| N° Fruit | **0.267** | 0.064 | -0.045 | 0.124 | 0.090 | 0.092 | 0.231 |
| Net photosynthesis (*P*_N_) | **0.246** | -0.013 | 0.245 | -0.108 | 0.172 | -0.092 | 0.089 |
| SLA | -0.187 | **0.275** | 0.121 | -0.200 | 0.160 | 0.086 | -0.008 |
| Total AsA | 0.226 | **-0.244** | -0.003 | -0.087 | -0.123 | -0.003 | 0.073 |
| Root/Shoot | 0.149 | **0.326** | -0.105 | 0.123 | 0.237 | -0.094 | 0.131 |
| Hydrogen peroxide (H2O2) | 0.204 | **0.290** | -0.033 | 0.072 | 0.035 | 0.058 | 0.000 |
| Chlorophyll B | -0.191 | 0.179 | **0.276** | 0.166 | 0.240 | -0.018 | 0.265 |
| Carotenoids | -0.218 | 0.186 | **0.230** | 0.121 | 0.138 | 0.064 | 0.165 |
| Reduced glutathione | -0.033 | -0.250 | **0.413** | 0.250 | 0.087 | -0.292 | -0.100 |
| Shoot FW | 0.205 | -0.106 | 0.196 | **0.340** | -0.234 | 0.139 | 0.189 |
| Ascorbate peroxidase (APX) | -0.136 | 0.094 | -0.361 | **0.409** | -0.076 | -0.299 | -0.021 |
| Quantum yield of PSII (Φ_PSII_) | 0.176 | 0.192 | 0.235 | **-0.362** | 0.025 | 0.030 | -0.225 |
| Water use efficiency (*A*_N_/*E*) | 0.245 | -0.073 | 0.110 | **-0.267** | 0.142 | -0.141 | -0.174 |
| Root FW | 0.222 | 0.217 | -0.020 | **0.223** | 0.101 | 0.167 | 0.011 |
| N° Leaf | 0.172 | 0.143 | 0.176 | 0.288 | **-0.429** | 0.178 | -0.235 |
| Reduced AsA | 0.070 | -0.239 | -0.192 | 0.153 | **0.603** | -0.099 | -0.185 |
| Chlorophyll A | -0.223 | 0.113 | 0.284 | -0.030 | 0.001 | **0.331** | -0.070 |
| Leaf Area | 0.019 | -0.384 | 0.041 | 0.067 | 0.238 | **0.412** | 0.070 |
| Peroxidases (POD) | -0.219 | -0.078 | -0.061 | 0.205 | 0.177 | **0.484** | -0.400 |
| Lipid peroxidation | 0.217 | 0.038 | -0.256 | -0.179 | 0.071 | 0.310 | **0.423** |
| Total glutathione | -0.107 | -0.327 | 0.278 | -0.006 | -0.045 | -0.116 | **0.336** |
| Catalase (CAT) | -0.231 | -0.163 | -0.184 | -0.053 | -0.127 | 0.153 | **0.308** |
| Boldface number indicate the most relevant traits for each principal components | | | | | |  |  |
