# Supplementary material for: The efficient physiological strategy of a novel tomato genotype to adapt to chronic combined water and heat stress
Source: Plant Biol (Stuttg). 2021 Oct 4;24(1):62–74. doi: 10.1111/plb.13339 (PMC9293464; doi:10.1111/plb.13339)
Supplement: Supplementary file 3 — Table S3. List of the 57 variants with a moderate and high impact effect on the protein structure of 47 genes. [file PLB-24-62-s003.docx]

**Table S3.** List of the 57 variants with a moderate and high impact effect on the protein structure of 47 genes.

| Gene | Private | Effect | Impact | Gene annotation |
| --- | --- | --- | --- | --- |
| Solyc01g008170 | E42 | missense_variant | MODERATE | Zinc finger transcription factor 5 |
| Solyc01g013910 | E42 | missense_variant | MODERATE | Endoribonuclease Dicer 2b |
| Solyc01g014520 | E42 | missense_variant | MODERATE | Receptor-like protein kinase |
| Solyc01g017640 | E42 | missense_variant&splice_region_variant | MODERATE | Unknown protein |
| Solyc01g028900 | E42 | missense_variant | MODERATE | 2-oxoisovalerate dehydrogenase subunit beta, mitochondrial |
| Solyc01g038231 | E42 | stop_gained | HIGH | Unknown protein |
| Solyc01g038231 | E42 | missense_variant | MODERATE | Unknown protein |
| Solyc01g038231 | E42 | missense_variant | MODERATE | Unknown protein |
| Solyc01g038231 | E42 | missense_variant | MODERATE | Unknown protein |
| Solyc01g056570 | E42 | missense_variant | MODERATE | Regulator of chromosome condensation |
| Solyc01g056890 | E42 | missense_variant | MODERATE | protein CURVATURE THYLAKOID 1A, chloroplastic-like |
| Solyc01g056890 | E42 | missense_variant | MODERATE | protein CURVATURE THYLAKOID 1A, chloroplastic-like |
| Solyc01g057703 | E42 | missense_variant | MODERATE | ATP-dependent DNA helicase |
| Solyc01g067790 | E42 | missense_variant | MODERATE | BRCT domain-containing DNA repair protein |
| Solyc01g068640 | E42 | missense_variant | MODERATE | AarF domain-containing protein kinase 4 |
| Solyc01g079380 | E42 | missense_variant | MODERATE | Transcription factor GRAS |
| Solyc01g079380 | E42 | missense_variant | MODERATE | Transcription factor GRAS |
| Solyc01g079380 | E42 | missense_variant | MODERATE | Transcription factor GRAS |
| Solyc01g079380 | E42 | missense_variant | MODERATE | Transcription factor GRAS |
| Solyc01g079380 | E42 | missense_variant | MODERATE | Transcription factor GRAS |
| Solyc01g080360 | E42 | missense_variant | MODERATE | AP-5 complex subunit mu |
| Solyc01g088640 | E42 | missense_variant | MODERATE | E3 ubiquitin ligase BIG BROTHER-related-like |
| Solyc01g161160 | E42 | missense_variant | MODERATE | Copia-type polyprotein |
| Solyc01g161350 | E42 | missense_variant&splice_region_variant | MODERATE | Tetratricopeptide repeat |
| Solyc01g163060 | E42 | missense_variant | MODERATE | cysteine-rich RECEPTOR-like kinase |
| Solyc01g163080 | E42 | missense_variant | MODERATE | Gag/pol polyprotein |
| Solyc02g086610 | E42 | splice_acceptor_variant&intron_variant | HIGH | Isocitrate dehydrogenase [NADP] |
| Solyc03g161560 | E42 | missense_variant | MODERATE | Protein REVEILLE 1 |
| Solyc04g024720 | E42 | missense_variant | MODERATE | Xyloglucan galactosyltransferase KATAMAR |
| Solyc05g013280 | LA3120 | missense_variant | MODERATE | Pseudomonas resistance |
| Solyc05g053650 | E42 | missense_variant | MODERATE | 26S proteasome non-ATPase regulatory subunit |
| Solyc07g005250 | E42 | missense_variant | MODERATE | Unknown protein |
| Solyc07g005530 | E42 | missense_variant | MODERATE | Ubiquitin carboxyl-terminal hydrolase 23 |
| Solyc07g017510 | E42 | missense_variant | MODERATE | 1-phosphatidylinositol-3-phosphate 5-kinase FAB1B |
| Solyc07g020735 | E42 | missense_variant | MODERATE | Unknown protein |
| Solyc07g021200 | E42 | missense_variant | MODERATE | Ribulose bisphosphate carboxylase large chain |
| Solyc07g021370 | E42 | stop_lost&splice_region_variant | HIGH | Unknown protein |
| Solyc07g021370 | E42 | missense_variant | MODERATE | Unknown protein |
| Solyc07g021540 | E42 | missense_variant | MODERATE | GRAM domain-containing protein |
| Solyc07g041100 | E42 | missense_variant | MODERATE | Unknown protein |
| Solyc07g053300 | E42 | missense_variant | MODERATE | ABC transporter G family member 10 |
| Solyc07g053340 | E42 | missense_variant | MODERATE | F-box protein At3g07870-like |
| Solyc07g053640 | E42 | disruptive_inframe_insertion | MODERATE | Arabinogalactan-protein |
| Solyc07g056020 | E42 | missense_variant | MODERATE | Translation initiation factor IF-2 |
| Solyc07g062930 | E42 | missense_variant | MODERATE | Ribosomal L |
| Solyc07g064400 | E42 | missense_variant | MODERATE | Aminotransferase-like, plant mobile domain-containing protein |
| Solyc07g065870 | E42 | missense_variant | MODERATE | Regulatory protein RecX |
| Solyc07g150121 | E42 | missense_variant | MODERATE | Serine/threonine-protein phosphatase 7 long form-like protein |
| Solyc11g005100 | E42 | missense_variant | MODERATE | NAD kinase |
| Solyc11g006420 | E42 | missense_variant | MODERATE | Pyrimidine 5'-nucleotidase |
| Solyc11g007010 | E42 | missense_variant | MODERATE | Proline-, glutamic acid-and leucine-rich protein |
| Solyc11g007280 | E42 | missense_variant | MODERATE | Pleiotropic drug resistance protein 2 |
| Solyc11g007370 | E42 | missense_variant | MODERATE | Glycosyltransferase |
| Solyc11g007370 | E42 | missense_variant | MODERATE | Glycosyltransferase |
| Solyc11g007700 | E42 | missense_variant | MODERATE | mRNA cap guanine-N7 methyltransferase 2 |
| Solyc11g007780 | E42 | missense_variant | MODERATE | SEC12-like protein |
| Solyc12g011400 | E42 | missense_variant | MODERATE | Pentatricopeptide repeat-containing protein, mitochondrial |
